# Satisfaction of Telehealth in Patients With Established Neuromuscular Disorders

**DOI:** 10.3389/fneur.2021.667813

**Published:** 2021-05-20

**Authors:** Sara Hooshmand, Junsang Cho, Shivangi Singh, Raghav Govindarajan

**Affiliations:** Department of Neurology, University of Missouri, Columbia, MO, United States

**Keywords:** neuromuscular disease, telehealth, satisfaction, survey, COVID-19

## Abstract

**Introduction/aims:** Determine established neuromuscular disease patients' satisfaction with telehealth during the COVID-19 pandemic.

**Methods:** We received 50 completed Utah telehealth satisfaction surveys from a cohort of 90 from April 2020 to June 2020. Returning neuromuscular disease patients rated seven aspects from 1 (strongly disagree) to 5 (strongly agree): Communication, timeliness of physician, picture quality, sound quality, protection of privacy, the comfort of the physical exam, the ease of healthcare, and whether patients would prefer “in-person” visits despite safety precaution. A favorable response was defined as a response of “Agree” or “Strongly Agree” to the survey questions. An independent *t*-test, Fisher's or chi-square test were used to compare demographic factors on outcomes for each survey question.

**Results:** The average age was 47.54 ± 20.63, 54% were female, 70% from rural areas, 60% had family present “webside,” and 14% had family present remotely. The majority of patients reported “Agree” or “Strongly Agree” to each survey question assessing their telehealth satisfaction, except for whether patients preferred in-person appointments. Demographic factors, including location and clinical diagnosis, did not influence survey responses.

**Discussion:** The vast majority of established neuromuscular disease patients responded favorably to their telehealth experience during the COVID-19 pandemic.

## Introduction

Telehealth (TH) is an electronic medium that provides health-related services and information (1). Due to the COVID-19 pandemic, TH provides an avenue which allows patients to not only receive medical care at geographical distances, but also minimizes the risk of viral transmission. TH is an established means of care in stroke, with systems delivering much-needed stroke expertise to hospitals and patients (2). Also, the integration of TH in pediatric neurology has also been viewed favorably in studies (2, 3). However, its role in other subspecialties within neurology has been less explored (4–7). In the neuromuscular setting, the majority of research to date has been focused on amyotrophic lateral sclerosis (ALS) (8). This study aims to determine established neuromuscular (NMD) patient satisfaction of telehealth during the COVID-19 pandemic.

## Materials and Methods

**Figure 1:**
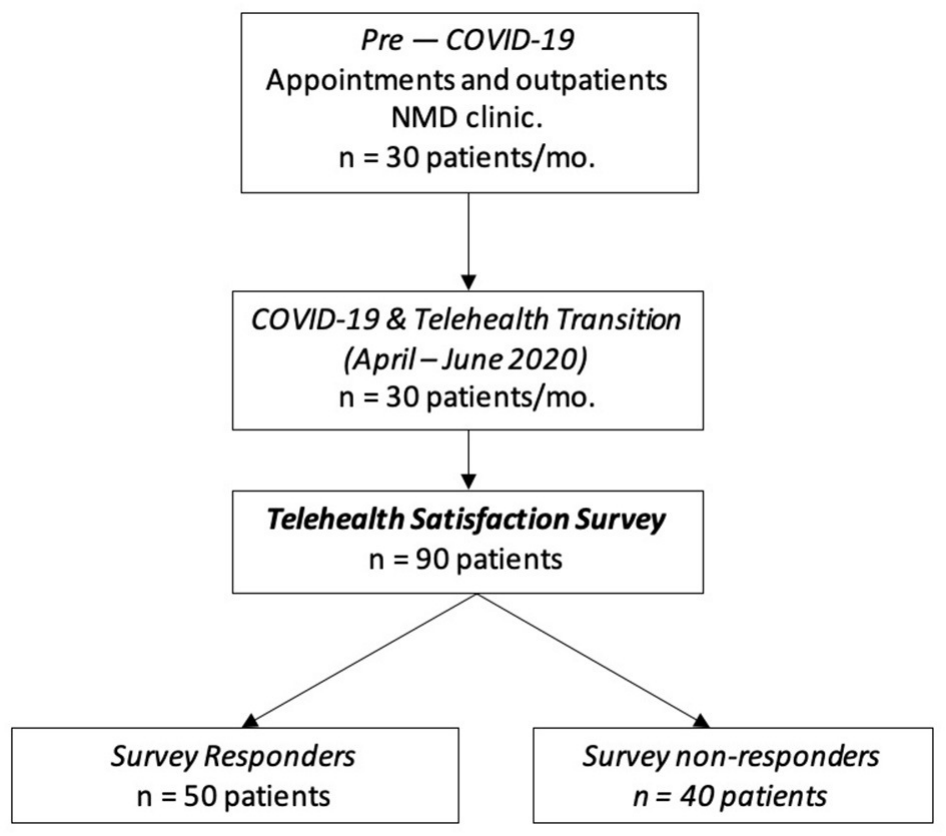
Schematic representation of patient selection. Data assessing patient satisfaction with video visits during the COVID-19 pandemic was collected between April and June 2020. All patients had an existing diagnosis of a neuromuscular disease (NMD) and were seen for return visits. A survey assessing the visits was sent to 90 patients. In that 90-person cohort, 50 were responders and 40 non-responders. Further analysis was done on the 50 individuals who responded.

This study was approved by the Institutional Review Boards (IRB) of the University of Missouri (Approval No. #2027587). Patients included in this study were men or women aged 15 and above, those with a neuromuscular diagnosis, and established patients at the University of Missouri NMD clinic with a minimum of 1 year follow-up. Due to the COVID-19 pandemic, from April to June 2020, the NMD clinic at University of Missouri was completely virtual. During this time, the Utah telehealth satisfaction surveys was provided to 90 patients, and we received 50 surveys from this cohort (Figure 1).

Patients were seen for follow-up visits *via* videoconferencing over a virtual healthcare platform, called Zoom for healthcare, and evaluated by author RG. Surveys about patient satisfaction with a virtual visit were then administered by the physician at the end of the visit (20% of the surveys) or mailed out by a nurse to the patients (80% of the surveys).

The Utah Telehealth Patient Satisfaction survey rated eight questions from 1 (strongly disagree) to 5 (strongly agree) (9). Questions 1–7 evaluated patients' satisfaction with telehealth and assessed the following components of the patients' virtual visit: communication, timeliness of physician, picture quality, sound quality, protection of privacy, the comfort of the physical exam, and ease of receiving telehealth. Question 8 assessed patients' preference for an “in-person” visits despite safety precautions and travel inconvenience. Lastly, in order to analyze for potential biases, we compared the results of the surveys that were administered by the physician vs. the surveys that were mailed in.

A favorable response to the survey questions was defined as responding “Agree” or “Strongly Agree” to questions 1–7, and “Disagree” or “Strongly Disagree” to question 8. An independent *t*-test, Fisher's or chi-square test were used to compare age and other clinical/demographic factors on favorable outcomes. *T*-tests were used to compare results of surveys administered by the physician vs. those mailed out. All statistical analysis was performed using SPSS Statistics version 27.

## Results

### Patient Characteristics

A total of 50 patient satisfaction surveys were analyzed. Patient demographic factors are described in Table 1. The patient sample included patients from a range 15–88 years old; average 47.54 ± 20.63. The majority were female (54%), and most patients were from rural locations (70%). The most common diagnoses included Myasthenia Gravis (20%), Amyotrophic Lateral Sclerosis (10%), and Charcot Marie Tooth Type 1A (8%) (Table 1).

**Table 1:** Demographic data of neuromuscular disease patients.

	***N* = 50**
Age (years)	
Mean ± SD	47.54 ± 20.63
Range	15 - 88
Sex [%, (*N*)]	
Male	46 (23)
Female	54 (27)
Ethnicities [%, (*N*)]	
Caucasian	88 (44)
African-American	8 (4)
Hispanic	4 (2)
Location [%, (*N*)]	
Rural	70 (35)
Urban	30 (15)
Family present webside [%, (*N*)]	
Yes	60 (30)
No	40 (20)
Family present remotely [%, (*N*)]	
Yes	14 (7)
No	86 (43)
Diagnoses [%, (*N*)]	
MG	20 (10)
ALS	10 (5)
CMT1A	8 (4)
BMD	6 (3)
MD1	6 (3)
LEMS	6 (3)
Other	44 (22)

*SD, Standard Deviation; MG, Myasthenia Gravis; ALS, Amyotrophic Lateral Sclerosis; CMT1A, Charcot Marie Tooth Type 1A; BMD, Becker Muscular Dystrophy; MD1, Myotonic Dystrophy Type 1; LEMS, Lambert-Eaton myasthenic syndrome*.

### Satisfaction Scores

The results of the Utah Telehealth Patient Satisfaction are outlined in Table 2. For each survey question, the majority of patients viewed telehealth favorably.

**Table 2:** Utah Telehealth Survey Responses by neuromuscular patients.

**Statement**	**Strongly agree**	**Agree**	**Neutral**	**Disagree**	**Strongly disagree**
1) I was able to communicate adequately with the specialist today, % (*N*)	46 (23)	54 (27)	0	0	0
2) The specialist was on time for the appointment, % (*N*)	50 (25)	50 (25)	0	0	0
3) The picture quality was good, % (*N*)	39 (19)	50 (25)	6 (3)	6 (3)	0
4) The sound quality was good, % (*N*)	28 (19)	50 (25)	6 (3)	4 (2)	2 (1)
5) My privacy and confidentiality were respected and protected during the consultation, % (*N*)	44 (22)	56 (28)	0	0	0
6) I was comfortable with the telehealth physical exam that was done, % (*N*)	40 (20)	54 (27)	4 (2)	2 (1)	0
7) Telehealth made it easier to get healthcare today, % (*N*)	50 (25)	44 (22)	6 (3)	0	0
8) Next time, I would prefer to see the specialist “in-person” despite the possible travel inconveniences, % (*N*)	2 (1)	0	64 (32)	32 (16)	2 (1)

For survey questions regarding effective communication, punctuality of physician, and maintaining privacy, all of the patients recorded either “Agree” or “Strongly Agree.” With respect to the picture and sound quality, 89 and 78% of patients reported their responses as either “Agree” or “Strongly Agree,” respectively. Moreover, 94% of patients reported either “Agree” or “Strongly Agree” when asked about the comfort of the physical exam done through telehealth. Lastly, all patients reported “Agree” or “Strongly Agree” when evaluating if their privacy and confidentiality were protected throughout the visit (Table 2). Question 8 on the survey assessed patients' preference to in-person visits by asking if patients would prefer to have seen the specialist in person, which 34% patients disagreed or strongly disagreed with this statement.

Clinical diagnosis and demographic factors had no significant effect on survey responses (Table 3). Lastly, survey results for those administered by the physician vs. those mailed out to patients were comparable (Table 4).

**Table 3:** Demographic factors on survey outcomes.

**Patient demographics**	**Outcomes**	***P*-values**
Age (years) mean ± SD		
Favorable	45.86 ± 20.59	0.12
Non-favorable	59.83 ± 17.86	
Sex (favorable cases) % (*N*)		
Male	91.3 (21/23)	0.67
Female	85.2 (23/27)	
Ethnicities (favorable cases) % (*N*)		
Caucasian	88.6 (39/44)	0.56
Other	83.3 (5/6)	
Location (favorable cases) % (*N*)		
Rural	88.6 (31/35)	0.99
Urban	86.7 (13/15)	
Family present webside (favorable cases) % (*N*)		
Yes	90.0 (27/30)	0.67
No	85.0 (17/20)	
Family present remotely (favorable cases) % (*N*)		
Yes	85.7 (6/7)	0.99
No	88.4 (38/43)	
Diagnoses (favorable cases) % (*N*)		
MG	90.0 (9/10)	0.99
Other	87.5 (35/40)	

*Favorable outcome defined as answering 4 (agree) or 5 (strongly agree) on all survey components*.

*SD, Standard Deviation; MG, Myasthenia Gravis; ALS, Amyotrophic Lateral Sclerosis; CMT1A, Charcot Marie Tooth Type 1A; BMD, Becker Muscular Dystrophy; MD1, Myotonic Dystrophy Type 1; LEMS, Lambert-Eaton myasthenic syndrome*.

**Table 4:** Average response ratings for survey administered by physician vs. mail.

**Survey component**	**(*N*) mean ± SD**	***P*-value**
Communication		
Physician	4.40 ± 0.52	0.68
Mail	4.48 ± 0.51	
Timeliness		
Physician	4.50 ± 0.53	0.99
Mail	4.50 ± 0.51	
Picture quality		
Physician	4.30 ± 0.67	0.67
Mail	4.18 ± 0.85	
Sound quality		
Physician	4.30 ± 0.68	0.63
Mail	4.15 ± 0.92	
Protection of privacy		
Physician	4.50 ± 0.53	0.68
Mail	4.43 ± 0.50	
Comfort of physical exam		
Physician	4.40 ± 0.52	0.67
Mail	4.30 ± 0.69	
Ease of healthcare		
Physician	4.40 ± 0.70	0.82
Mail	4.45 ± 0.60	
Preference “in-person”		
Physician	2.50 ± 0.71	0.31
Mail	2.73 ± 0.60	

## Discussion

Although telestroke has become more widespread since 1999, utilization telehealth in the outpatient setting for neuromuscular disorders is less commonly adopted (10). TH provides many advantages over in-person clinic visits such as decreased transportation time and cost, reduction of patient, caregiver childcare burden, and increased access to specialized care (8). Despite these advantages, neuromuscular clinics were slower to adopt TH likely due to the need for in person neurological exam. Additionally, NMDs can be complicated by respiratory issues and need for durable medical equipment, which may better suit an in-person multidisplicinary clinic (11). However, the COVID-19 pandemic necessitated a transition to TH as a predominant healthcare delivery model, which was aided by the relaxation of prior regulatory limitations (12). Due to the increased utilization of TH, it was important to understand NMD patients' satisfaction.

Our results revealed that returning NMD patients viewed communication, timeliness, picture quality, sound quality, privacy/confidentiality, the comfort of the physical exam, and ease of receiving telehealth favorably. Notably, there was no significant difference in survey response when considering patient's demographics or diagnosis.

While our results are promising, this study is limited in its small sample size and the lack of a control group. There is no standardized TH patient satisfaction survey for NMD patients. Although the Utah telehealth satisfaction surveys captures various aspects of a medical visit, limitations include generalizability and recall bias. Despite these limitations, the survey captures aspects of a telehealth encounter that ultimately provide valuable insight in understanding patients' satisfaction during the COVID-19 pandemic. Further studies with larger patient sample sizes and in-person clinic control groups are needed to further support our results.

There were potential biases involved for surveys administered in the presence of the physician. Fortunately, our results show that average responses were comparable to survey mailed out to patients. In addition, there was a potential bias involved in the response rate of our survey 55% (50/90). However, based on a study by Susan H Spooner revealed that a survey response of >50% did not bias the survey despite there being non-responders, making the chance of non-response bias low in this study (13).

Lastly, although the majority of patients viewed the sound/picture quality favorably, we believe broadband width could be a potential culprit for the five rural patients who disagreed with this question on the survey. Also, additional training for physician to talk slower may also improve patients' experiences with telehealth.

Despite these limitations, our results are consistent with prior studies investigating neurological patients' satisfaction with TH. Specifically, research has demonstrated patients with established ALS view TH appointments favorably, with the most common conveyed sentiment being that TH removes the burden of travel (8). Similarly, previous studies on telehealth satisfaction scores in Parkinson disease, another chronic neurological condition, has demonstrated favorably satisfaction of TH (14, 15).

In April 2020, the American Academy of Neurology also provided guidelines for the integration of TH in NMD (11). Among the discussed outpatient visits, follow-up appointments for NMD were given a “high utility/appropriateness” designation. Included in this category were return visits for stable peripheral neuropathy, myasthenia gravis, myositis, and other stable neuropathies/myopathies. Accordingly, our study reinforces the utility of telehealth in the evaluation of returning NMDs. Our study reveals that established NMD patients have a favorable view of TH during the COVID-19 pandemic.

## Data Availability Statement

The original contributions presented in the study are included in the article/supplementary material, further inquiries can be directed to the corresponding author/s.

## Ethics Statement

The studies involving human participants were reviewed and approved by Institutional Review Boards (IRB) of the University of Missouri (Approval No. #2027587). Written informed consent from the participants' legal guardian/next of kin was not required to participate in this study in accordance with the national legislation and the institutional requirements.

## Author Contributions

SH, JC, SS, and RG contributed to the design and editing of the manuscript. JC performed the statistical analysis. SH wrote the first draft of the manuscript. JC and SS wrote sections of the manuscript. All authors contributed to the manuscript revisions, read, and approved the submitted version.

## Conflict of Interest

The authors declare that the research was conducted in the absence of any commercial or financial relationships that could be construed as a potential conflict of interest.

## Abbreviations

TH: telehealth
NMD: neuromuscular diseases
ALS: amyotrophic lateral sclerosis
IRB: Institutional Review Boards.
